# Docosahexaenoic Acid Modulates NK Cell Effects on Neutrophils and Their Crosstalk

**DOI:** 10.3389/fimmu.2020.570380

**Published:** 2020-10-05

**Authors:** Kirstine Nolling Jensen, Sunnefa Yeatman Omarsdottir, Margret Sol Reinhardsdottir, Ingibjorg Hardardottir, Jona Freysdottir

**Affiliations:** ^1^ Faculty of Medicine, Biomedical Center, University of Iceland, Reykjavik, Iceland; ^2^ Department of Immunology, Landspitali—The National University Hospital of Iceland, Reykjavik, Iceland; ^3^ Center for Rheumatology Research, Landspitali—The National University Hospital of Iceland, Reykjavik, Iceland; ^4^ Faculty of Pharmaceutical Sciences, University of Iceland, Reykjavik, Iceland

**Keywords:** natural killer cells, neutrophils, docosahexaenoic acid, apoptosis, CD47, NKp46, phagocytosis

## Abstract

Natural killer (NK) cells and neutrophils engage in crosstalk that is important in inflammation and likely also for resolution of inflammation. NK cells activate neutrophils and induce their infiltration to the inflamed sites but may also influence their apoptosis and their subsequent efferocytosis by macrophages. Several studies indicate that docosahexaenoic acid (DHA) can inhibit NK cell cytotoxicity but the effects of DHA on the ability of NK cells to engage in crosstalk with neutrophils and affect their functions have not been described. This study explored the kinetics of the effects of NK cells and NK cells pre-treated with DHA on neutrophil surface molecule expression and apoptosis, as well as the ability of NK cells to affect other neutrophil functions. In addition, the study explored the effects of neutrophils on NK cell phenotype and function. Primary NK cells were pre-incubated with or without DHA, then stimulated and co-cultured with freshly isolated neutrophils. When co-cultured with NK cells, neutrophils had higher expression levels of CD11b and CD47; secreted more IL-8, IL-1ra, and CXCL10; had increased phagocytic ability; and their apoptosis was increased early after initiation of the co-culture while dampened at a later time-point. Pre-incubation of NK cells with DHA attenuated NK cell-induced upregulation of CD11b and CD47 on neutrophils, had minor effects on NK cell induction of cytokine/chemokine secretion or their phagocytic ability. Neutrophils also affected the function of NK cells, lowering the frequency of NKp46^+^ and CXCR3^+^ NK cells and increasing the concentrations of IFN-γ, TNF-α, and GM-CSF in the co-cultures. Pre-incubation of NK cells with DHA further decreased the frequency of NKp46^+^ NK cells in the co-culture with neutrophils and decreased the concentrations of IFN-γ, CCL3 and GM-CSF. These findings indicate that NK cells have mostly pro-inflammatory effects on neutrophils and that DHA can attenuate some of these pro-inflammatory effects. Neutrophils had both anti- and pro-inflammatory effects on NK cells. When NK cells had been pre-treated with DHA, the anti-inflammatory effects were increased and some of the pro-inflammatory effects attenuated. Overall, the results suggest that DHA may lead to a more anti-inflammatory microenvironment for NK cell and neutrophil crosstalk.

## Introduction

Natural killer (NK) cells are cytotoxic lymphocytes best known for their ability to target aberrant cells without prior sensitization (1). They are potent producers of cytokines and chemokines, such as IFN-γ, GM-CSF, TNF-α, and CCL3 (2). Neutrophils are innate cells that readily infiltrate inflamed sites and exert their functions through phagocytosis, cytotoxicity, extracellular traps, and secretion of a wide array of anti-microbial compounds, cytokines, and chemokines, such as IL-8, CXCL10, and IL-1ra (3–5).

NK cells and neutrophils engage in crosstalk and can modulate activation, infiltration, and survival of each other (6). One indication of NK cells being able to activate neutrophils is that they induce neutrophil expression of the integrin CD11b. This has been suggested to occur through NK cell production of GM-CSF (7, 8). That GM-CSF produced by NK cells has been shown to potentiate several chronic inflammatory disorders (9) may indicate involvement of NK cell-neutrophil crosstalk in these diseases. NK cells recruit neutrophils to inflammatory sites through several mechanisms, including the CCL3-CCR5 signaling axis (2, 10). Expression of CD47 on neutrophils is also implicated in inducing their transmigration in both bacterial and fungal infections (11–13). Furthermore, low CD47 expression on neutrophils has been associated with enhanced phagocytosis of anergic and apoptotic cells by macrophages and hence is regarded as being anti-inflammatory (14).

NK cells induce neutrophil apoptosis in fungal infections (15) through NKp46- and/or Fas-dependent mechanisms (16) and upregulation of MHC class I expression on neutrophils is associated with higher susceptibility to NK cell-induced apoptosis (17). On the contrary, two independent studies have shown that NK cells inhibit neutrophil apoptosis *in vitro* (7, 8). NK cells also play a role in modulating neutrophil reactive oxygen species (ROS) production, enhancing ROS production only when the neutrophils receive a low-grade stimulation (7, 8). NK cell ability to enhance neutrophil phagocytosis is thought to occur through a cell-to-cell mediated mechanism (7). However, their induction of neutrophil phagocytosis of *Candida albicans* as well as their ability to enhance fungicidal activity of neutrophils is through a mechanism yet to be described (18). Not only can NK cells affect neutrophil function, but neutrophils can also affect NK cell function. Neutrophils can act as a cellular source of IL-18 that in collaboration with IL-12 activates NK cells (19) and stimulates NK cell production of IFN-γ, TNF-α and GM-CSF. Neutrophil production of ROS induces NK cell apoptosis, primarily in the CD56^low^ subset (20, 21) and lowers their expression of NKp46 and thereby inhibits their cytotoxic function (22).

Omega-3 polyunsaturated fatty acids (PUFAs) have anti-inflammatory effects and affect both NK cells and neutrophils. Their effects on inflammation are partly because they are incorporated into cellular membranes at the expense of the omega-6 PUFA arachidonic acid (23, 24). Arachidonic acid is the substrate for pro-inflammatory lipid mediators, such as prostaglandins, thromboxane, leukotrienes, and lipoxins (25). On the other hand, the omega-3 PUFAs eicosapentaenoic acid and docosahexaenoic acid (DHA) are substrates for specialized pro-resolution mediators (SPMs), such as resolvins, protectins, and maresins, that drive resolution of inflammation (26, 27). Dietary omega-3 PUFAs inhibit NK cell cytotoxicity (28, 29) and thereby impair resistance to influenza in mice by suppressing NK cell cytotoxicity (30). In addition, the SPM Resolvin E1 enhances NK cell infiltration into inflamed tissues through their receptor ChemR23 (31), leading to the suggestion that NK cells actively contribute to resolution of inflammation (32). Our group showed that dietary fish oil enhanced the resolution phase of inflammation in antigen-induced peritonitis and led to an early peak in NK cell numbers compared to that in mice fed a control diet (33). We subsequently showed that depletion of NK cells in this model resulted in an increase in neutrophil infiltration to the inflamed site with the inflammation remaining unresolved for at least 24 h (34). These findings suggest that NK cells are pivotal players in limiting neutrophil infiltration to inflammatory sites and inducing resolution of inflammation. In the current study, we hypothesized that NK cells modulate neutrophil function, phenotype, and survival, that neutrophils might also affect NK cell phenotype and function, and that this crosstalk could be modulated by DHA. The results provide an insight into the kinetics of NK cell and neutrophil crosstalk, confirming that NK cells have mostly pro-inflammatory effects on neutrophils and that neutrophils affect NK cell phenotype and function. It also demonstrates that pre-incubating NK cells with DHA modulates the effects of NK cells on neutrophils on the NK cells in an anti-inflammatory manner.

## Materials and Methods

### Preparation of Docosahexaenoic Acid

Docosahexaenoic acid (DHA) was obtained from Cayman Chemical (Michigan, USA) as peroxidase free, in single-use ampules. DHA was dried down under nitrogen and resuspended in dimethyl sulfoxide (DMSO, Sigma-Aldrich, Germany) and flushed with nitrogen. Aliquots of DHA were stored at −80°C until needed. Before use, DHA was resuspended in RPMI 1640 medium (Gibco, Thermo Fisher Scientific, Massachusetts, USA), enriched with 10% fetal bovine serum (FBS, Gibco) and penicillin/streptomycin (Pen/Strep, Gibco) (complete RPMI medium) to a concentration of 2 mM and incubated at room temperature for 1 h to allow binding of DHA to albumin.

### NK Cell Isolation and Culture

Peripheral blood mononuclear cells (PBMCs) were isolated from buffy coats obtained from healthy volunteers at the Icelandic Blood Bank (permission # 06-068-V1). Buffy coats were diluted in phosphate buffered saline (PBS), layered over Histopaque-1077 (Sigma-Aldrich), centrifuged and the PBMC layer collected. NK cells were negatively isolated from PBMCs using an NK cell isolation kit (Miltenyi Biotec, Germany) following the manufacturer’s directions. The purity of the NK cells was determined by flow cytometry and was ~93%. NK cells were cultured in 48-well culture plates (Nunc, Thermo Fisher Scientific) in complete RPMI medium at a density of 1 × 10^6^ cells/ml. DHA was added at a final concentration of 50 µM (DHA-NK cells). Equal volume of DMSO was added to the cultures as a control (C-NK cells) with the final concentration of DMSO being 0.06%. The plates were incubated for 18 h at 37°C, 5% CO_2_ and 95% humidity to allow incorporation of DHA into the cell membranes, before the cells were stimulated with IL-2 (2 ng/ml), IL-12 (2 ng/ml), and IL-15 (10 ng/ml) (all from R&D Systems, Bio-Techne, United Kingdom).

### Neutrophil Isolation and Co-Culture With NK Cells

Neutrophils were isolated from fresh EDTA venous blood of healthy volunteers (permission # 06-068-V1) and incubated for 30 min at room temperature. Histopaque-1077 (Sigma-Aldrich) was carefully layered onto Histopaque-1119 (Sigma-Aldrich). EDTA venous blood was layered onto the Histopaques, centrifuged and the granulocyte-rich layer collected. Remaining erythrocytes were pelleted with 3% dextran (Sigma-Aldrich) and lysed in ACK lysis buffer. The purity of the neutrophils was determined by flow cytometry and was ~98%. Neutrophils were resuspended to a density of 2 × 10^6^ cells/ml in complete RPMI medium with IL-2, IL-12, and IL-15 and added to the NK cell cultures at a 1:2 ratio of NK cells and neutrophils. The cytokines added did not affect the phenotype or function of the neutrophils when cultured alone in control studies. The co-cultures were incubated at 37°C, 5% CO_2_, and 95% humidity for up to 24 h before the cells were harvested, pelleted, stained for surface molecules, and evaluated by flow cytometry. Supernatants were collected, aliquoted and kept at −80°C until cytokine and chemokine concentrations were measured by ELISA.

### Reactive Oxygen Species Production

Following co-culture of NK cells and neutrophils for 16.5 h, 10 µM 2′,7′-dichloroflourescein diacetate (DCFDA, Abcam, United Kingdom) was added to the wells. The cells were incubated for further 90 min at 37°C, 5% CO_2_ and 95% humidity. Cells were harvested, washed and ROS production was determined by flow cytometry using a Sony SH800 flow cytometer (Sony Biotechnology, United Kingdom). Results are presented as percent positive cells and cells without added DCFDA served as a negative staining control.

### Phagocytosis Assay

After co-culturing NK cells and neutrophils for 4 h, heat-inactivated, FITC-labelled *E. coli* (Abcam) (5 µl) were added to the wells. Cells were incubated for additional 2 h at 37°C, 5% CO_2_ and 95% humidity before being harvested, washed, and evaluated by flow cytometry using a Sony SH800 flow cytometer. Neutrophils not receiving FITC-labelled *E. coli* served as a negative control. Results are presented as percent positive cells compared to the negative control.

### ELISA

Concentrations of TNF-α, IFN-γ, IL-8 (CXCL8), CXCL10, IL-1ra, GM-CSF, and CCL3 in cell culture supernatants were determined using DuoSet ELISA kits (R&D Systems).

### Flow Cytometry

Cells were harvested after 3, 6, 12, 18, or 24 h of co-culture, pelleted and washed. Prior to staining, Fc-receptors were blocked by incubating the cells with 2% heat-inactivated mix of normal human serum and normal mouse serum (AbD Serotec, Bio-Rad, United Kingdom) and 5% TruStain FcX™ (BioLegend, California, USA) and stained for 20 min on ice. NK cells were stained with monoclonal fluorochrome-labeled antibodies against CD3 (OKT3, BioLegend), CD56 (CMSSB, eBioscience, Thermo Fisher Scientific), CD16 (3G8, BioLegend), CXCR3 (G025H7, BioLegend), and NKp46 (9E2, BioLegend. Neutrophils were stained with monoclonal fluorochrome-labeled antibodies against CD11b (M1/70, BioLegend), CD16a (CB16, BioLegend), CD47 (miap301, BioLegend), and CD62L (DREG-56, BioLegend). Appropriate isotype controls were used to determine positive staining. Apoptotic cells were stained using a FITC Annexin V apoptosis detection kit with propidium iodide (BioLegend) following the manufacturer’s instructions. Following washing, cells were fixed in 2% paraformaldehyde and kept at 4°C until they were evaluated on a Navios EX flow cytometer (Beckman Coulter, Indianapolis, USA) or a Sony SH800 flow cytometer. Live cells were gated based on their forward and side scatter. NK cells were defined as CD3^-^CD56^+^ lymphocytes and neutrophils were defined as CD16a^+^CD62L^+^CD49d^–^ granulocytes (see **Supplementary Figure 1**). Cells were analyzed using the Kaluza Analysis Software (Beckman Coulter).

### Statistical Analysis

Results are presented as means ± standard error of the mean (SEM). The n indicated in each figure legend refers to the number of independent cell donors, not technical replicates. All data presented are an average from a minimum of three independent experiments. Outliers were identified using the Grubbs’ method with an α of 0.05, omitting only one outlier per group, if appropriate. Groups were compared using one-way or two-way ANOVA. Difference between groups was regarded as significant when the p-value < 0.05. All statistical analysis was carried out in GraphPad Prism 8 (GraphPad Software, California, USA).

## Results

### DHA Attenuates NK Cell Induction of CD47 and CD11b Expression on Neutrophils

**Figure 1:**
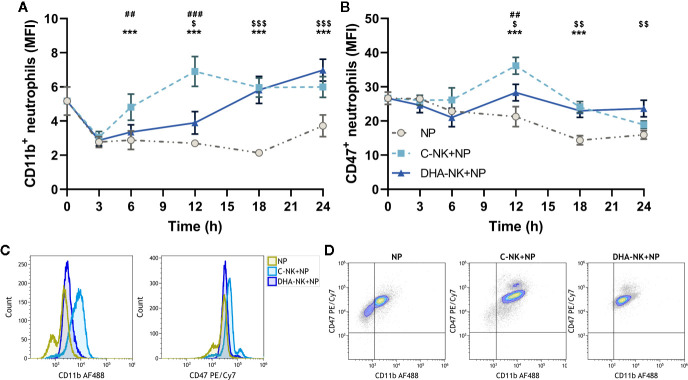
The effects of NK cells and NK cells pre-incubated with DHA on neutrophil expression of CD11b **(A)** and CD47 **(B)**. Neutrophils were culture alone (NP) or with NK cells that had been pre-incubated for 18 h without (C-NK+NP) or with 50 µM of docosahexaenoic acid (DHA-NK+NP) and then stimulated with IL-2 (2 ng/ml), IL-12 (2 ng/ml), and IL-15 (10 ng/ml). The cells were cultured together for 0, 3, 6, 12, 18, and 24 h. Expression levels were determined by flow cytometry and are presented as mean fluorescence intensity (MFI). Representative overlay histograms of CD11b and CD47 expression levels at 12 h **(C)**. Representative dot plots of expression levels of CD11b and CD47 at 12 h **(D)**. Positive gating was determined with appropriate isotype controls. Data are shown as mean ± SEM; * indicates difference between C-NK+NP and NP, $ difference between DHA-NK+NP and NP, and # difference between DHA-NK+NP and C-NK+NP. One symbol indicates p < 0.05, two symbols p < 0.01, and three symbols p < 0.001. n = 6 (independent donors), collected in three independent experiments.

Expression of CD47 and CD11b on neutrophils is pivotal for neutrophil transmigration and defense against both bacterial and fungal infections (11, 35). Previous studies suggest that NK cells upregulate CD11b expression on neutrophils through their GM-CSF production (7). **Figure 1A** shows that NK cells cultured without DHA (C-NK cells) rapidly upregulate CD11b expression on neutrophils after 6 h in co-culture. The CD11b upregulation was maintained throughout the 24 h co-culture period with the highest expression observed at 12 h (**Figures 1A, C**). Co-culturing neutrophils with C-NK cells also enhanced their expression levels of CD47 at 12 and 18 h with the highest expression observed at 12 h (**Figures 1B, C**). Interestingly, co-culturing neutrophils with NK cells pre-incubated with DHA (DHA-NK cells) delayed their upregulation of CD11b and expression levels of CD11b did not reach the same levels as when the neutrophils were co-cultured with C-NK cells until at 18 h (**Figures 1A, C**). Co-culturing neutrophils with DHA-NK cells also attenuated neutrophil upregulation of CD47 at 12 h (**Figures 1B, C**) but enhanced it in a similar manner as co-culturing neutrophils with C-NK cells at 18 h (**Figure 1B**). All neutrophils expressed CD47 but the proportion of neutrophils expressing CD11b increased when co-cultured with C-NK cells or DHA-NK cells (**Figure 1D**). A small population of neutrophils expressing higher levels of CD47 appeared when they were co-cultured with either C-NK cells or DHA-NK cells (**Figure 1D**).

### DHA Does Not Affect NK Cell Induction of Neutrophil Production of IL-8, IL-1ra, or CXCL10

**Figure 2:**
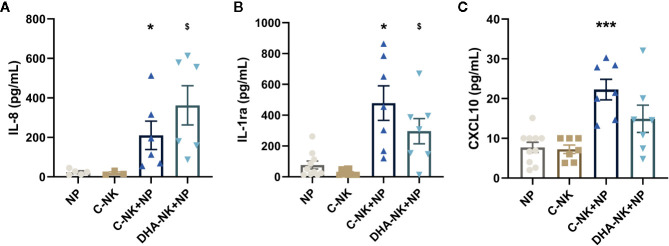
The effects of NK cells and NK cells pre-incubated with DHA on neutrophil production of IL-8 **(A)**, IL-1ra **(B)**, and CXCL10 **(C)**. Neutrophils (NP) and NK cells (C-NK) were cultured alone or together after the NK cells had been pre-incubated for 18 h without (C-NK+NP) or with 50 µM docosahexaenoic acid (DHA-NK+NP) and then stimulated with IL-2 (2 ng/ml), IL-12 (2 ng/ml), and IL-15 (10 ng/ml). The cells were cultured together for 18 h. Cytokine concentrations in supernatants were determined by ELISA and are presented as pg/ml. Data are shown as mean ± SEM; * indicates difference between C-NK+NP and NP, and $ difference between DHA-NK+NP and NP. One symbol indicates p < 0.05 and three symbols p < 0.001. One outlier was removed from the NP group in A and B and one outlier in the C-NK group in C. n = 5 - 11 (except for C-NK in A, n = 3), collected in 4 independent experiments. In **Supplementary Figure 2** all outliers are included.

Neutrophils produce high levels of IL-8 and CXCL10 to potentiate inflammatory responses (4, 5) but mediate anti-inflammatory responses by producing IL-1ra (3). Higher levels of IL-8, IL-1ra, and CXCL10 were present in supernatants when neutrophils and C-NK cells were cultured together compared with that when either cell type was cultured alone (**Figures 2A–C**). Co-culturing neutrophils with DHA-NK cells showed a slightly higher average concentration of IL-8 (**Figure 2A**) and slightly lower concentrations of IL-1ra and CXCL10 (**Figures 2A, B**) than when co-culturing them with C-NK cells, but the differences were not statistically significant and it is doubtful that they have biological significance.

### DHA Dampens Further NK Cell-Induced Lowering of Neutrophil Apoptosis

**Figure 3:**
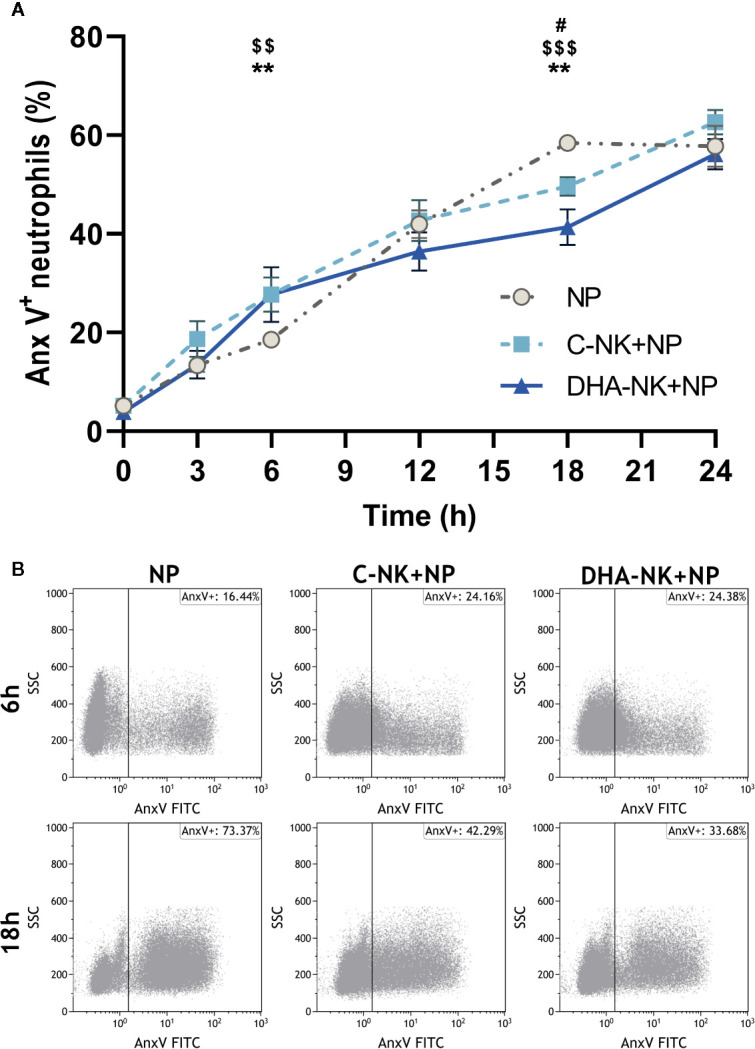
The effects of NK cells and NK cells pre-incubated with DHA on neutrophil apoptosis. Neutrophils were cultured alone (NP), or with NK cells that had been pre-incubated for 18 h without (C-NK+NP) or with 50 µM docosahexaenoic acid (DHA-NK+NP) and then stimulated with IL-2 (2 ng/ml), IL-12 (2 ng/ml), and IL-15 (10 ng/ml). The cells were cultured together for 0, 3, 6, 12, 18, and 24 h. Apoptosis was determined by flow cytometric analysis of annexin V (Anx V) binding to neutrophils and are presented as Anx V^+^ neutrophils **(A)**. Representative dot plots of Anx V binding to neutrophils after 6 and 18 h of co-culture **(B)**. Positive gating was determined with an unstained control. Data are shown as mean ± SEM; * indicates difference between C-NK+NP and NP, $ difference between DHA-NK+NP and NP, and # difference between DHA-NK+NP and C-NK+NP. One symbol indicates p < 0.05, two symbols p < 0.01, and three symbols p < 0.001. n = 8 (independent donors), collected in 4 independent experiments.

**Figure 4:**
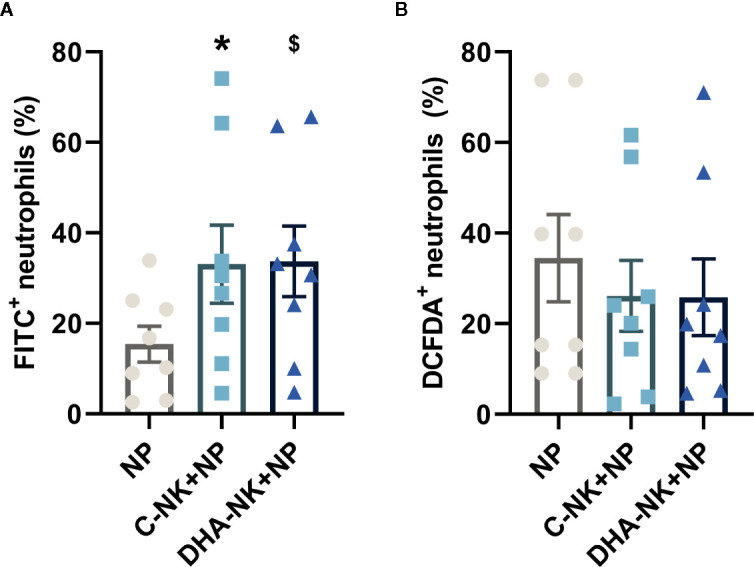
The effects of NK cells and NK cells pre-incubated with DHA on neutrophil phagocytosis **(A)** and reactive oxygen species production **(B)**. Neutrophils were cultured alone (NP) or with NK cells that had been pre-incubated for 18 h without (C-NK+NP) or with 50 µM docosahexaenoic acid (DHA-NK+NP) and then stimulated with IL-2 (2 ng/ml), IL-12 (2 ng/ml), and IL-15 (10 ng/ml). The cells were cultured together for 6 h. Phagocytosis was determined by flow cytometric analysis of engulfed FITC-labelled *E. coli* and presented as percent of the total granulocyte population **(A)**. Reactive oxygen species production was measured by DCFDA conversion to the highly fluorescent 2′,7′-dichlorofluorescein, determined by flow cytometry and presented as percent of the total neutrophil population **(B)**. Positive gating was determined by appropriate untreated controls. Data are shown as mean ± SEM; * indicates difference between C-NK+NP and NP and $ difference between DHA-NK+NP and NP. One symbol indicates p < 0.05. n = 8 (independent donors), collected in three independent experiments.

NK cells have been shown to modulate neutrophil survival by either inhibiting (7, 8) or inducing (15, 16) their apoptosis. Additionally, NK cells enhance neutrophil phagocytosis and ROS production through undetermined mechanisms (7, 8). In the present study, addition of C-NK cells to neutrophils led to a slight increase in their apoptosis after 6 h of co-culture (**Figures 3A, B**). However, when prolonging the co-culture to 18 h, C-NK cells seemed to delay their apoptosis as it had reached similar levels as in the control group at 24 h (**Figures 3A, B**). DHA-NK cells did not affect neutrophil apoptosis differently from C-NK cells at 6 h but had more of a dampening effect on neutrophil apoptosis at 18 h (**Figures 3A, B**). Neutrophil phagocytosis was enhanced when they were co-cultured with C-NK cells for 6 h (**Figure 4A**) but their production of ROS was not affected by co-culturing them with C-NK cells (**Figure 4B**), contrary to previous findings (8). Pre-incubating the NK cells with DHA did not alter their enhancement of neutrophil phagocytosis nor their lack of effect on neutrophil ROS production (**Figures 4A, B**).

### Neutrophils Modulate NKp46 Expression on NK Cells Pre-Incubated With DHA

**Figure 5:**
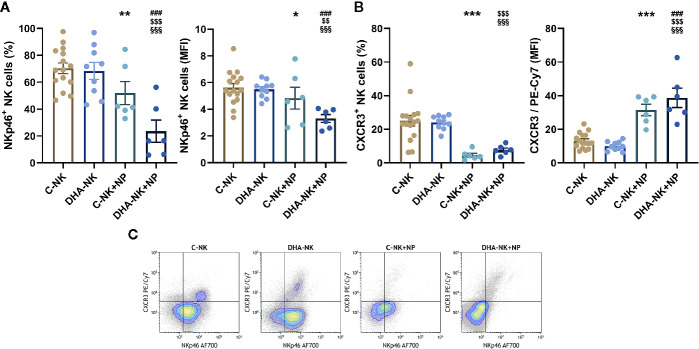
Effects of neutrophils on expression of NKp46 **(A, C)** and CXCR3 **(B, C)** on NK cells pre-incubated with DHA or not. NK cells were pre-incubated for 18 h in the absence (C-NK) or presence (DHA-NK) of 50 µM docosahexaenoic acid and then stimulated with IL-2 (2 ng/ml), IL-12 (2 ng/ml), and IL-15 (10 ng/ml) and cultured further for 6 h alone or with neutrophils (C-NK+NP and DHA-NK+NP). Expression levels were determined by flow cytometry and are presented either as percent positive cells of the total NK cell population or as mean fluorescent intensity (MFI) of the positive cell population **(A, B)**. Positive gating was determined with appropriate isotype controls. Data are shown as mean ± SEM; * indicates difference between C-NK+NP and C-NK, § difference between DHA-NK and DHA-NK+NP, $ difference between DHA-NK+NP and C-NK, and # difference between DHA-NK+NP and C-NK+NP. One symbol indicates p < 0.05, two symbols p < 0.01, and three symbols p < 0.001. n = 6–15 (independent donors), collected in at least three independent experiments.

NK cells induce neutrophil apoptosis through an NKp46- and/or Fas-dependent mechanism (16). CXCR3 mediates NK cell migration to draining lymph nodes during inflammation (2). In this study, when co-cultured with neutrophils a lower proportion of C-NK cells expressed NKp46 and expression levels of NKp46 were also lower than when the NK cells were cultured alone (**Figure 5A**). When DHA-NK cells were co-cultured with neutrophils the proportion of the NK cells expressing NKp46 and expression levels of NKp46 were still lower than when C-NK cells were co-cultured with neutrophils (**Figure 5A**). The proportion of NK cells expressing CXCR3 was also lower when the NK cells were co-cultured with neutrophils as compared with when the NK cells were cultured alone, regardless of whether the NK cells had been pre-incubated with DHA or not (**Figure 5B**). A small population of NK cells expressing CXCR3 and high levels of NKp46 (NKp46^hi^CXCR3^+^) was present on NK cells cultured alone but mostly disappeared when the NK cells were co-cultured with neutrophils (**Figure 5C**). The tiny population of NKp46^+^CXCR3^hi^ NK cells present following co-culture with neutrophils (**Figure 5C**) resulted in the higher expression levels of CXCR3 in the co-cultures with neutrophils (**Figure 5B**). When NK cells pre-treated with DHA were cultured alone their expression of NKp46 and CXCR3 was not different from that of untreated NK cells (**Figures 5A–C**).

### Pre-Incubation of NK Cells With DHA Reduces Neutrophil-Induced NK Cell Production of IFN-γ and GM-CSF

**Figure 6:**
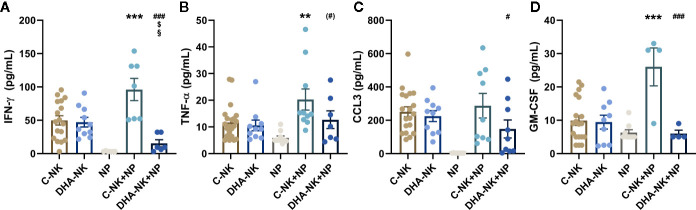
The effects of neutrophils on production of IFN-γ **(A)**, TNF-α **(B)**, CCL3 **(C)**, and GM-CSF **(D)** in co-cultures with NK cells pre-incubated with DHA or not. NK cells were pre-incubated for 18 h in the absence (C-NK) or presence (DHA-NK) of 50 µM docosahexaenoic acid and then stimulated with IL-2 (2 ng/ml), IL-12 (2 ng/ml), and IL-15 (10 ng/ml) and cultured with or without neutrophils for further 18 h (C-NK+NP and DHA-NK+NP). Neutrophils were also cultured alone for 18 h (NP). Cytokine concentrations were determined by ELISA and are presented as pg/ml. Data are presented as means ± SEM; * indicates difference between C-NK+NP and C-NK, § difference between DHA-NK and DHA-NK+NP, $ difference between DHA-NK+NP and C-NK, and # difference between DHA-NK+NP and C-NK+NP. One symbol indicates p < 0.05, two symbols p < 0.01, and three symbols p < 0.001. Symbol in parenthesis indicates p = 0.05–0.1. One outlier was removed in the NP group in A and B, one outlier was removed in the C-NK+NP group in B and D and one outlier was removed in the DHA-NK+NP group in A. n = 4–21 (independent donors), collected in at least three independent experiments. In **Supplementary Figure 3** all outliers are included.

Previous studied have shown that neutrophils can enhance NK cell production of IFN-γ, TNF-α and GM-CSF (6, 19) and that NK cells can modulate neutrophil function and migration through their expression of CCL3 (6). Culturing C-NK cells with neutrophils increased their secretion of IFN-γ, TNF-α and GM-CSF compared with that when the C-NK cells were cultured alone (**Figures 6A, B, D**). The concentration of IFN-γ was lower in co-cultures of DHA-NK cells and neutrophils than in co-cultures of C-NK cells and neutrophils and had a tendency towards being lower than when C-NK cells were cultured alone (**Figure 6A**). In addition, lower concentrations of CCL3 and GM-CSF were present in co-cultures of DHA-NK cells and neutrophils compared with that in co-cultures of C-NK cells and neutrophils (**Figures 6C, D**). Pre-treatment of NK cells with DHA did not affect their secretion of the cytokines (**Figures 6A–D**). Neutrophils cultured alone produced very low levels of IFN-γ, TNF-α and CCL3 (**Figures 6A–C**) but produced GM-CSF at similar levels as untreated NK cells cultured alone (**Figure 6D**).

## Discussion

Omega-3 PUFAs affect inflammation and its resolution (26, 27). Whether they affect the crosstalk between NK cells and neutrophils, a crosstalk important for inflammation and its resolution, has not been previously described. In the present study, DHA attenuated the effects of NK cells to enhance neutrophil expression of the pro-inflammatory surface molecules CD11b and CD47. DHA did not affect NK cell induction of neutrophil phagocytosis nor their ROS production. On the other hand, DHA enhanced the pro-survival (anti-apoptotic) effect NK cells have on neutrophils late in their co-culture. Pre-incubation of NK cells with DHA also modulated the effects neutrophils had on the NK cells, enhancing their ability to decrease NK cell expression of NKp46 and CXCR3, but decreasing their secretion of IFN-γ, CCL-3, and GM-CSF. Hence, the results indicate that DHA has mostly anti-inflammatory effects on the crosstalk between NK cells and neutrophils.

NK cells enhanced neutrophil expression of CD11b and CD47 and their phagocytosis, similar to what has been shown by Costantini et al. and Bhatnagar et al. (7, 8). Neutrophil expression of CD11b and CD47 is important for their transmigration to inflamed sites (12, 35), therefore, NK cell induction of neutrophil expression of these surface molecules may render them more capable of transmigrating to the inflamed sites. NK cell production of CCL3 may also promote extravasation (36). Pre-incubation of NK cells with DHA, in the present study, reduced the ability of the NK cells to induce neutrophil expression of both CD11b and CD47 and decreased their production of CCL3 in co-cultures with neutrophils, thereby possibly tempering NK cell ability to induce migration of neutrophils to inflamed sites. The lowered expression of CD47 caused by pre-incubating NK cells with DHA may also induce neutrophil efferocytosis by macrophages as low levels of CD47 have been shown to promote this way of removal of apoptotic neutrophils (14). Neutrophil expression of CD11b and CD47 has also been indicated to be important for neutrophil phagocytosis of pathogens (12, 35) and NK cell induction of neutrophil expression of CD11b and CD47 may be involved in increased neutrophil phagocytosis seen when co-culturing C-NK cells with neutrophils in the present study. However, decreased CD11b and CD47 expression on neutrophils co-cultured with NK cells pre-incubated with DHA was not accompanied by a reduction in the ability of NK cells to induce neutrophil phagocytosis, which was maintained as high as when the neutrophils were incubated with NK cells cultured in the absence of DHA.

NK cells produce cytokines that activate neutrophils, including IFN-γ and TNF-α (6), and neutrophils produce a wide array of cytokines and chemokines, including IL-8, that can induce chemotaxis of several innate immune cells (37). In the present study, TNF-α concentration was higher in co-cultures of NK cells and neutrophils than when the cells were cultured alone, which could have led to the increase in IL-8 production by the neutrophils when cultured with NK cells. Furthermore, neutrophil production of the anti-inflammatory IL-1ra was increased when the neutrophils were co-cultured with NK cells, possibly through increased NK cell TNF-α and GM-CSF production (38). Pre-incubation of NK cells with DHA suppressed their production of IFN-γ when co-cultured with neutrophils. This diminished production of IFN-γ by DHA pre-treatment of NK cells did not lead to a decrease in IL-8 production, suggesting that IL-8 production was induced by another mechanism.

NK cells induce and inhibit neutrophil apoptosis depending on the stimulus and timing as described in several studies. NK cells have been shown to induce neutrophil apoptosis through NKp46 and/or Fas-signaling or by diminishing MHC class I expression on neutrophils (16, 17). In addition, NK cells are suggested to either inhibit or increase neutrophil apoptosis through a GM-CSF-mediated mechanism (7, 8, 17). In the present study, we examined the kinetics of the effects of NK cells on neutrophil apoptosis, to shed light on these contradicting results. NK cells induced neutrophil apoptosis after 6 h in co-culture, similar to that seen in the studies by Bernson et al. and Thorén et al. also investigating apoptosis at early time-points (16, 17). By contrast, prolonged co-culture (18 h) of NK cells and neutrophils suppressed neutrophil apoptosis, comparable to that shown by Bhatnagar et al. and Costantini et al. (7, 8). NK cell induction of neutrophil apoptosis at 6 h, in the present study, is not likely to be mediated by NK cell expression of NKp46 as suggested by Thorén et al. (16) as co-culture with neutrophils decreased NKp46 expression and pre-incubation of NK cells with DHA decreased NKp46 expression even more. Neither is it likely that the effects of NK cells on neutrophil apoptosis in the present study were mediated by a GM-CSF-mediated mechanism as co-culturing NK cells with neutrophils increased their GM-CSF production but when the NK cells had been pre-treated with DHA their GM-CSF production was reduced to even lower than that when they were cultured alone. Surprisingly, pre-incubating NK cells with DHA enhanced NK cell-induced suppression of apoptosis at 18 h leading to increased survival of the neutrophils. These results indicate not only the potential of NK cells to prevent resolution of inflammation but also that pre-incubation with DHA could enhance this anti-resolution function of the NK cells.

According to Mair et al. NKp46^hi^CXCR3^+^ cells are potent responders in inflammation (39). However, in the present study, when NK cells were co-cultured with neutrophils the NKp46^hi^CXCR3^+^ population that was present when NK cells were cultured alone more or less disappeared, suggesting that these NK cells were less likely to migrate to draining lymph nodes, as described by Martín-Fontecha et al. (40), and subsequently to induce an inflammatory response.

In summary, the results from this study show that pre-treatment of NK cells with DHA attenuated NK cell ability to induce upregulation of CD11b and CD47 expression on neutrophils. This may indicate that DHA can diminish NK cell ability to promote neutrophil migration to inflamed sites. However, pre-treatment with DHA enhanced the pro-survival effect of NK cells on neutrophils, late in the co-culture, indicating that DHA could increase NK cell ability to hamper neutrophil removal from the inflamed site. Pre-treatment of NK cells with DHA also diminished NK cell expression of activation molecules and production of several pro-inflammatory cytokines in co-cultures with neutrophils. Our findings indicate that DHA may attenuate some of the pro-inflammatory effects NK cells have on neutrophils, as well as increase the anti-inflammatory and attenuate some of the pro-inflammatory effects neutrophils have on NK cells. Overall, the results suggest that DHA may lead to a more anti-inflammatory microenvironment for NK cell and neutrophil crosstalk.

## Data Availability Statement

The raw data supporting the conclusions of this article will be made available by the authors, without undue reservation.

## Ethics Statement

The studies involving human participants were reviewed and approved by The National Bioethics Committee, Iceland. The patients/participants provided their written informed consent to participate in this study.

## Author Contributions

KJ, IH, and JF designed the research. KJ, SO, and MR conducted the research and obtained the data. KJ, SO, MR, IH, and JF analyzed the data. IH and JF supervised the study. KJ, IH, and JF drafted the manuscript. All authors contributed to the article and approved the submitted version.

## Funding

This study was funded by the Icelandic Research Fund (# 173973051), The University of Iceland Research Fund (both project and doctoral), Landspitali University Hospital Research Fund, and the Memorial Fund of Helga Jonsdottir and Sigurlidi Kristjansson.

## Conflict of Interest

The authors declare that the research was conducted in the absence of any commercial or financial relationships that could be construed as a potential conflict of interest.

The handling editor declared a past co-authorship with two of the authors, JF and IH.
