# Supplementary figures and images for: Docosahexaenoic Acid Modulates NK Cell Effects on Neutrophils and Their Crosstalk
Source: Front Immunol. 2020 Oct 5;11:570380. doi: 10.3389/fimmu.2020.570380 (PMC7573488; doi:10.3389/fimmu.2020.570380)

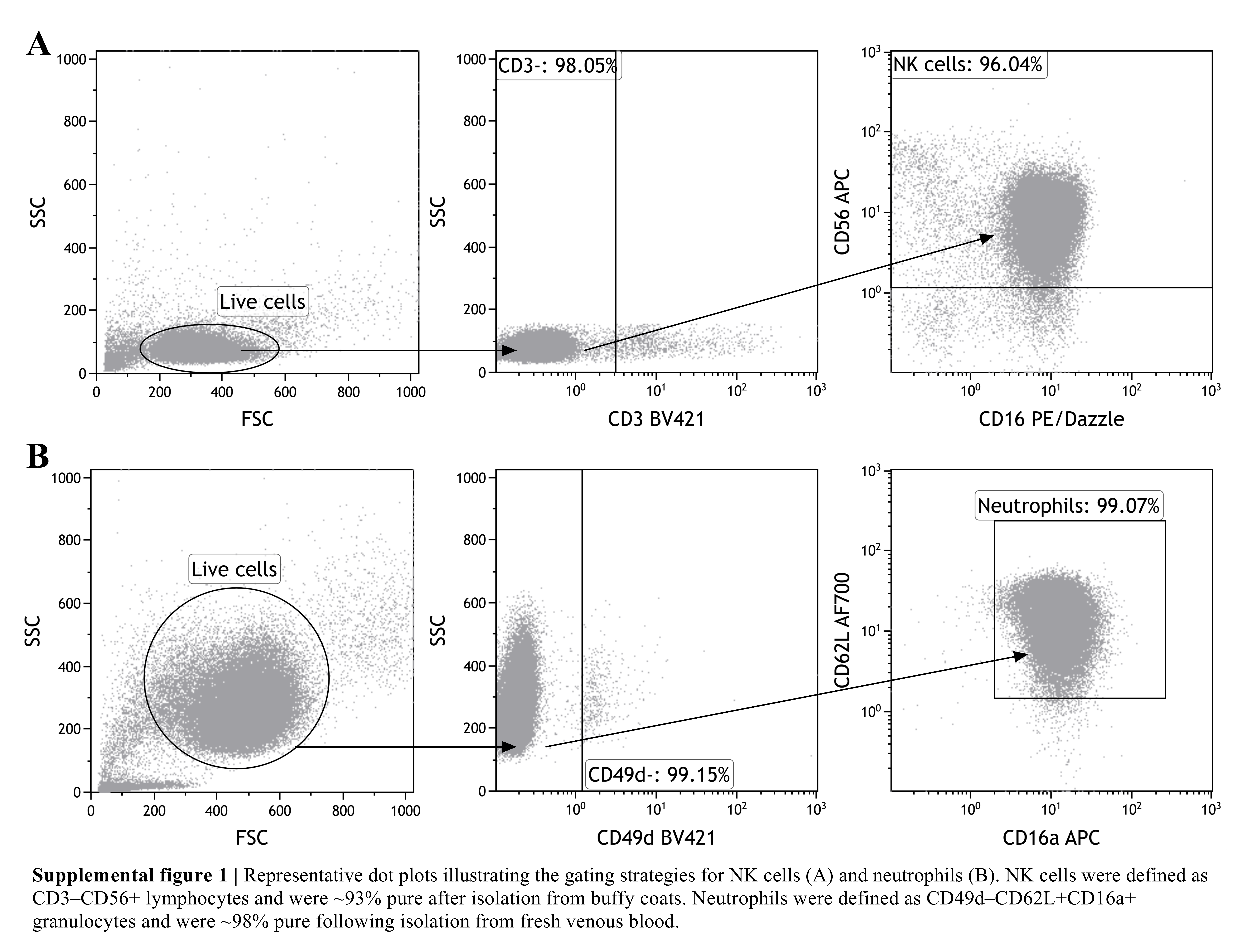

Supplement: Supplementary file 1 [file Image_1.tif]

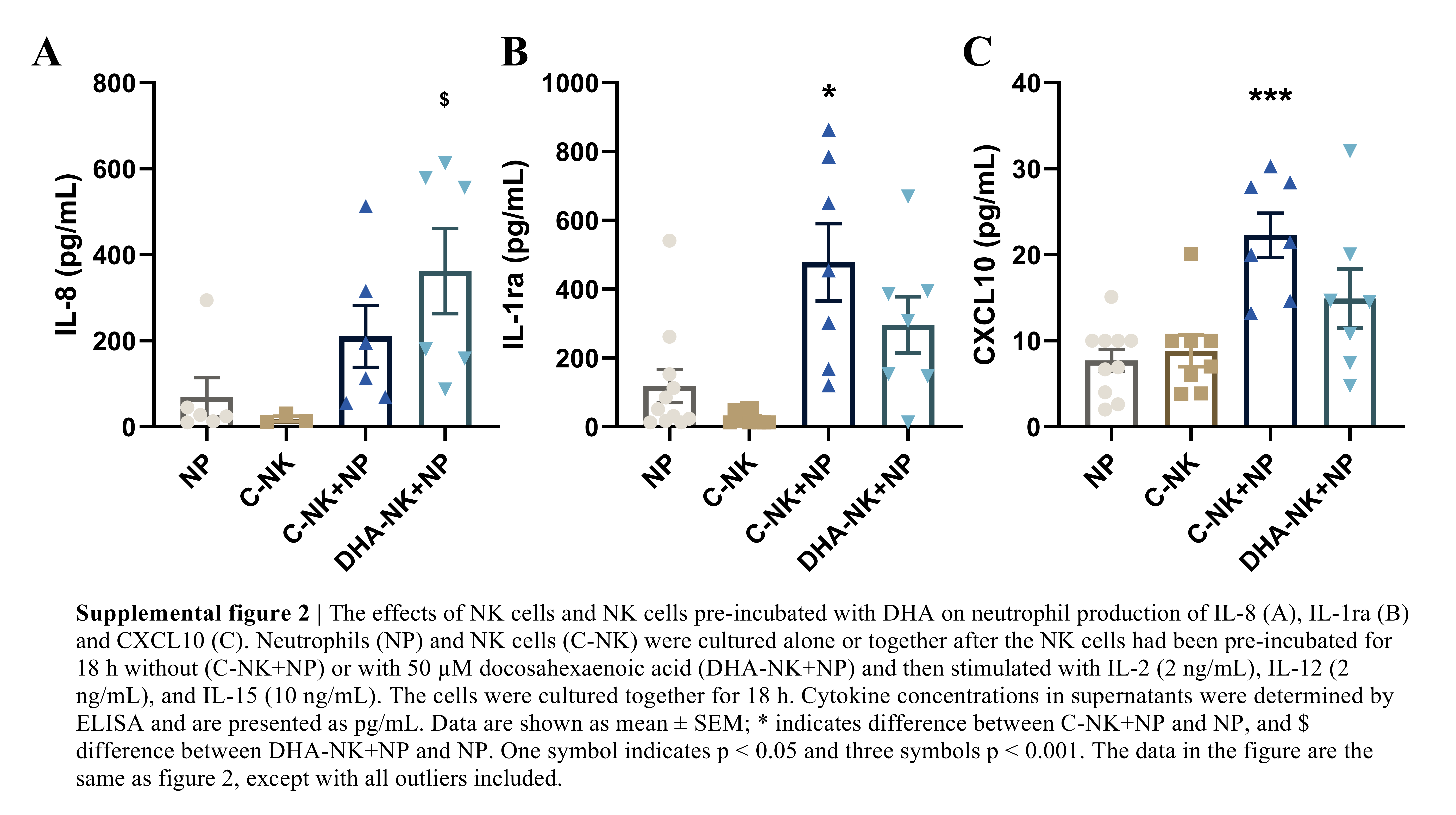

Supplement: Supplementary file 2 [file Image_2.tif]

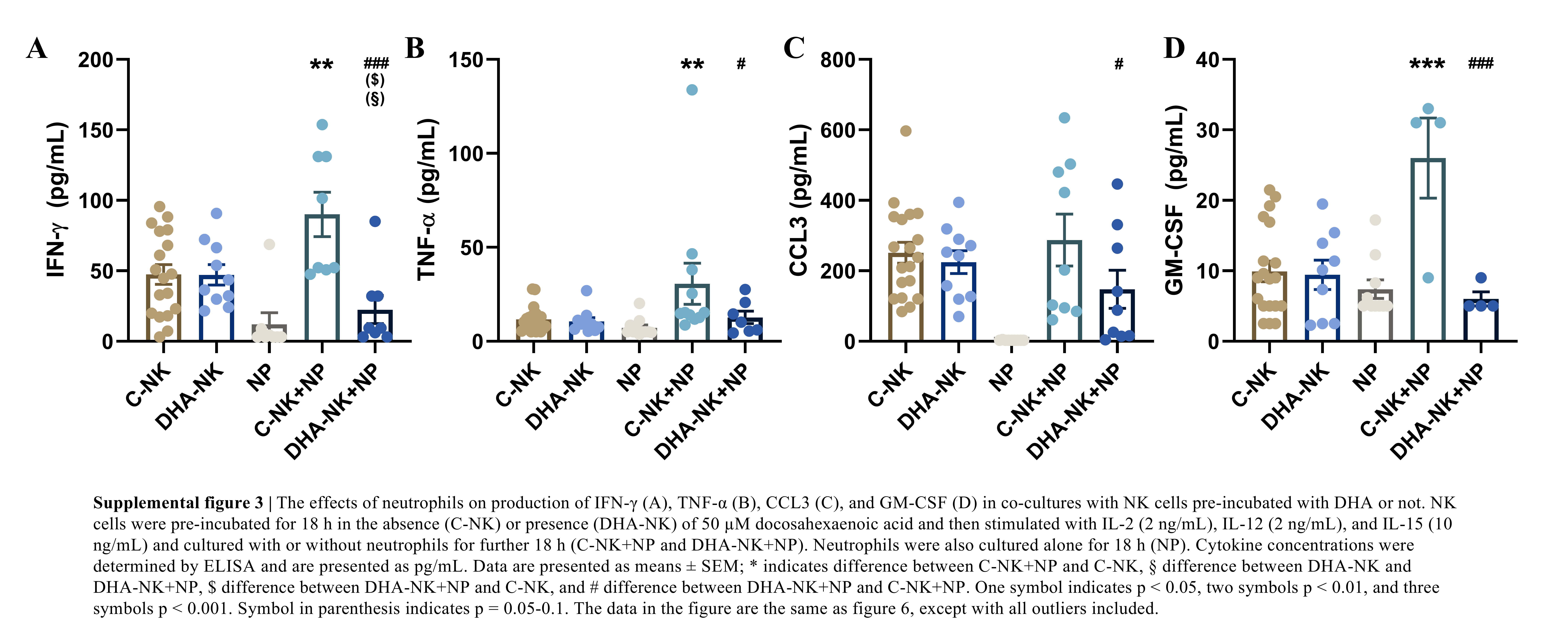

Supplement: Supplementary file 3 [file Image_3.tif]
